# Detection of periodontal bone loss patterns and furcation defects from panoramic radiographs using deep learning algorithm: a retrospective study

**DOI:** 10.1186/s12903-024-03896-5

**Published:** 2024-01-31

**Authors:** Sevda Kurt-Bayrakdar, İbrahim Şevki Bayrakdar, Muhammet Burak Yavuz, Nichal Sali, Özer Çelik, Oğuz Köse, Bilge Cansu Uzun Saylan, Batuhan Kuleli, Rohan Jagtap, Kaan Orhan

**Affiliations:** 1 Faculty of Dentistry, Department of Periodontology, Eskisehir Osmangazi University, Eskisehir, 26240 Turkey; 2 Division of Oral and Maxillofacial Radiology, Department of Care Planning and Restorative Sciences, University of Mississippi Medical Center School of Dentistry, Jackson, MS USA; 3 Faculty of Dentistry, Department of Oral and Maxillofacial Radiology, Eskisehir Osmangazi University, Eskisehir, Turkey; 4 Faculty of Science, Department of Mathematics and Computer Science, Eskisehir Osmangazi University, Eskisehir, Turkey; 5 Faculty of Dentistry, Department of Periodontology, Recep Tayyip Erdogan University, Rize, Turkey; 6 Faculty of Dentistry, Department of Periodontology, Dokuz Eylül University, İzmir, Turkey; 7 Faculty of Dentistry, Department of Orthodontics, Eskisehir Osmangazi University, Eskisehir, Turkey; 8 Division of Oral and Maxillofacial Radiology, Department of Care Planning and Restorative Sciences, University of Mississippi Medical Center School of Dentistry, Jackson, MS USA; 9 Faculty of Dentistry, Department of Oral and Maxillofacial Radiology, Ankara University, Ankara, Turkey

**Keywords:** Panoramic radiography, Artificial intelligence, Periodontitis, Dentistry

## Abstract

**Background:**

This retrospective study aimed to develop a deep learning algorithm for the interpretation of panoramic radiographs and to examine the performance of this algorithm in the detection of periodontal bone losses and bone loss patterns.

**Methods:**

A total of 1121 panoramic radiographs were used in this study. Bone losses in the maxilla and mandibula (total alveolar bone loss) (*n* = 2251), interdental bone losses (*n* = 25303), and furcation defects (*n* = 2815) were labeled using the segmentation method. In addition, interdental bone losses were divided into horizontal (*n* = 21839) and vertical (*n* = 3464) bone losses according to the defect patterns. A Convolutional Neural Network (CNN)-based artificial intelligence (AI) system was developed using U-Net architecture. The performance of the deep learning algorithm was statistically evaluated by the confusion matrix and ROC curve analysis.

**Results:**

The system showed the highest diagnostic performance in the detection of total alveolar bone losses (AUC = 0.951) and the lowest in the detection of vertical bone losses (AUC = 0.733). The sensitivity, precision, F1 score, accuracy, and AUC values were found as 1, 0.995, 0.997, 0.994, 0.951 for total alveolar bone loss; found as 0.947, 0.939, 0.943, 0.892, 0.910 for horizontal bone losses; found as 0.558, 0.846, 0.673, 0.506, 0.733 for vertical bone losses and found as 0.892, 0.933, 0.912, 0.837, 0.868 for furcation defects (respectively).

**Conclusions:**

AI systems offer promising results in determining periodontal bone loss patterns and furcation defects from dental radiographs. This suggests that CNN algorithms can also be used to provide more detailed information such as automatic determination of periodontal disease severity and treatment planning in various dental radiographs.

## Background

Periodontitis is a chronic inflammatory disease that is characterized by damage to the supporting tissues of the teeth and can result in tooth loss if not controlled [1]. This common disease in society is associated with many systemic diseases in the body as well as dental problems such as chewing loss [2, 3]. In this respect, early diagnosis and treatment planning are very important in periodontitis cases [2, 3].

Radiographic evaluations play an important role in the diagnosis of periodontitis in addition to clinical periodontal evaluations such as probing pocket depth, attachment loss, and gingival recession [4]. Intraoral radiographs such as periapical radiography and bitewing radiography are commonly used to determine periodontal status [4–6]. Panoramic radiographs, which is one of the extraoral dental radiographic techniques, have advantages such as working with low-dose radiation providing quick and easy radiological imaging, and also helping in determining general dental problems and periodontal conditions [6–8]. In addition, these radiographs allow the observation of bone loss patterns (such as horizontal bone loss, vertical bone loss, and furcation defects) in periodontitis cases [9]. In periodontitis cases, it is very important to determine the bone loss patterns and bone morphology to make an appropriate treatment plan and achieve successful results [10, 11]. Horizontal bone loss occurs when the bone supporting the tooth melts at the same levels on all tooth-related surfaces (mesial, distal, vestibular/buccal, lingual/palatal). In this type of bone defect, the alveolar crest levels around the teeth are resorbed parallel to the line that should be in the healthy periodontium and are positioned more apically [12–14]. Vertical bone losses are bone losses that occur obliquely and angularly in the interdental region [12–14]. In multi-rooted teeth, furcation defects occur when the alveolar bone between the tooth roots is also affected by periodontal disease. In furcation defects, both diagnosis and treatment planning become much more complex and difficult for dentists [15]. The correct estimation of bone loss patterns enables the correct treatment selection and facilitates the work of both patients and dentists. For example, flap surgery and/or resective surgical periodontal therapy may be preferred rather than regenerative treatment in horizontal bone losses, while regenerative treatment may be an alternative method in vertical bone losses [14]. According to the current classification of periodontal and peri-implant diseases and conditions developed in 2018, the detection of periodontal bone loss patterns is very important for determining disease stages [16]. In this classification system, Stage I and II periodontitis are associated with the presence of horizontal bone loss, while the presence of vertical bone losses and furcation defects in Stage III and IV periodontitis is noted [16, 17]. Current academic studies using image processing and machine learning technologies and aiming at automatic periodontal disease classification have pointed out the determination of periodontal bone loss patterns as one of the main classification criteria [17, 18]. Therefore, the determination of periodontal bone loss pattern is of clinical importance in making the current periodontal disease classification complete and accurate [17, 18].

Various studies about artificial intelligence (AI) have been carried out in dentistry, following the use of AI systems in the medical field for disease diagnosis and treatment planning [19, 20]. In these studies, it is seen that AI systems are used for the determination of many pathologies such as dental caries, apical lesions, tooth numbering, and root fractures on two-dimensional (2D) radiographs [21–24]. The main purpose of using AI in dentistry practice is to automatically detect pathologies, diseases, or anatomical structures and determine disease severity. While achieving these goals, it also provides additional benefits such as preventing situations that may be overlooked due to the physician’s inexperience, intensity, and fatigue, ensuring early diagnosis of diseases, and recording patient data more regularly in the digital environment [6, 20].

There are several studies for evaluating bone losses and periodontal problems using AI systems [3, 6, 18, 25–30]. However, as far as we know, there is no AI-based study aiming to determine the periodontal bone loss patterns using the segmentation method. This study aimed to examine the performance of a convolutional neural network (CNN)-based AI system in the detection of periodontal problems such as horizontal bone losses, vertical bone losses, and furcation defects on panoramic radiographs by segmentation method.

## Methods

### Study design

All of the images were obtained from the radiology archive of the Eskişehir Osmangazi University Faculty of Dentistry for this retrospective study. Ethics board approval was obtained from the Non-interventional Clinical Research Ethics Committee of Eskişehir Osmangazi University before starting the study (decision number: 2019 − 227). The guidelines of the Declaration of Helsinki were followed throughout all phases of the study.

### Data selection

All images used in the study were obtained with the same radiography device (Planmeca Promax 2D, Planmeca, Helsinki, Finland) with the following parameters: 68 kVp, 16 mA, and 13 s. All images were selected from images of individuals over the age of 18, without paying attention to age and gender differences. In radiographs, 2 mm apical of the imaginary line passing through the cemento-enamel junction of the teeth was accepted as the healthy periodontium level [31–33]. Cases whose alveolar bone crest was coronal to this imaginary line were considered to have healthy periodontium and were excluded from the study. Images of periodontitis cases of different ages and genders were re-evaluated in terms of the criteria stated below: ‘1. The images with dense artifacts, 2. The images with low quality due to patient positioning error or patient movement during acquisition, 3. The images of patients who received orthognathic treatment, 4. The images of patients with bone metabolism disorders, 5. The images of patients with unusual alveolar bone morphology (bone pathologies due to conditions such as cysts and tumors), 6. The images of patients with cleft lip and palate, 7. The images of poor quality and blurred images in the alveolar bone region due to conditions such as dental crowding, 8. The images of patients with metal restorations that cause artifacts to complicate periodontal diagnosis’. Images that meet these criteria, which would make diagnosis difficult and disrupt the standardization of the study, were also excluded from the study.

### Ground truth

All data that were compatible with the inclusion criteria were uploaded into the CranioCatch labeling module (CranioCatch, Eskisehir, Turkey) in an anonymized manner. Initially, intra-observer and inter-observer compatibility were analyzed for the 4 observers (3 periodontists: SKB, MBY, NS, and 1 oral maxillofacial radiologist: İŞB) who planned to carry out the labeling to ensure labeling standardization. For intra-observer evaluation, the observers labeled all the parameters to be evaluated in this study in line with the criteria below on 10 radiographs with the segmentation method. Each observer repeated this procedure 1 week later. A computer program (Python version 3.6.1, Python Software Foundation) and NumPy library were used and the splicing of the segmented fields was done and Intersection over Union (IoU) values were calculated. When it was decided that there was intra-observer and inter-observer aggregation for labeling, all data were evaluated by the same observers according to the same criteria, and the labeling of the relevant parameters was completed.

**Fig. 1:**
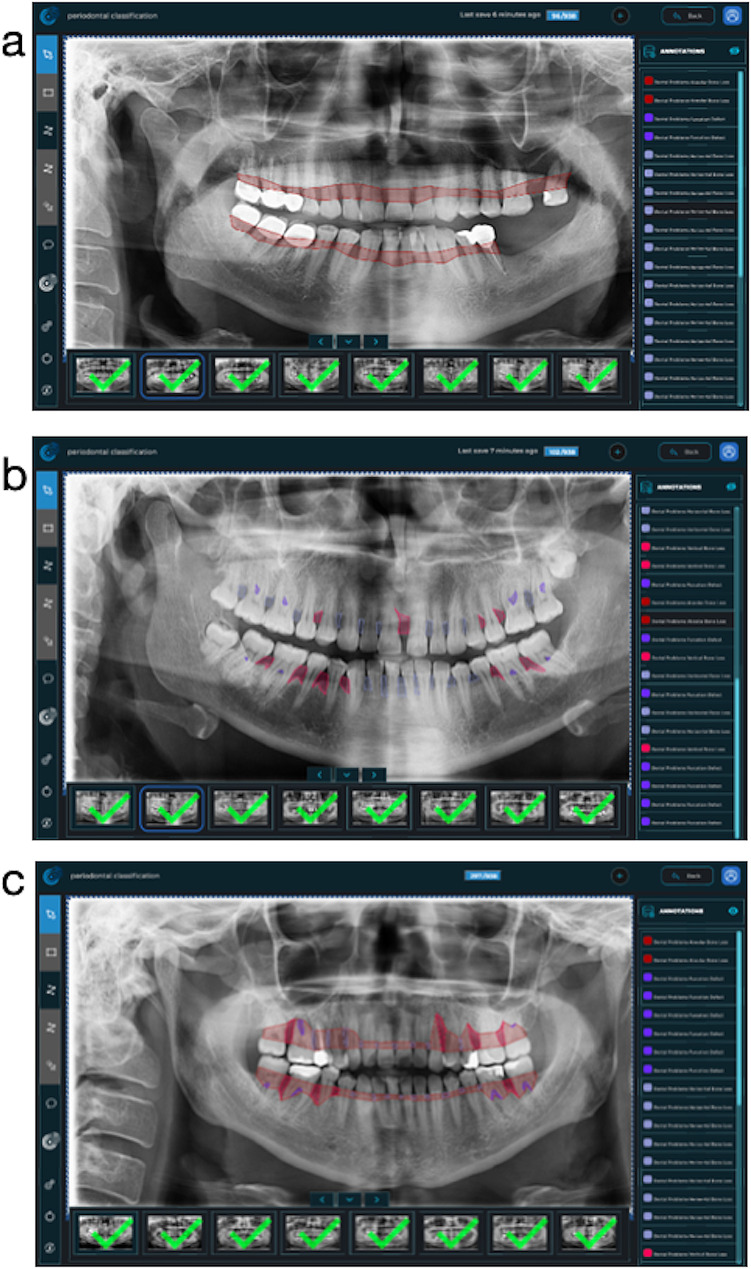
Images of the labeling module. (**a**) Labeling of a patient’s total alveolar bone loss; (**b**) Labeling of a patient’s interdental bone loss (red: vertical, blue: horizontal, purple: furcation); (**c**) Labeled version of all data of a patient

The criteria taken into consideration during the labeling phase were:

*For total alveolar bone loss*: The unit of this parameter was the jaw (maxilla and mandible). Two separate segmentation processes were performed for the maxilla and mandible in all panoramic radiographs. While performing the segmentation, the root parts of the teeth without bone support and the interdental regions following these areas were included in the label area. Initially, a segmentation line was created from the cemento-enamel junction of all teeth. After that, the segmentation line was combined to follow the line on the distal surfaces of the most distal teeth and the border of the bone crests in the relevant jaw. In Fig. 1-a, an example image was presented to better understand the path followed when labeling this parameter. (Fig. 1-a)

In the next stage, the interdental regions were evaluated one by one. A separate labeling process was made for these areas, taking into account the bone loss patterns (in the form of horizontal and vertical bone loss) (Fig. 1-b). The units of these parameters were the interdental region.

*For horizontal bone losses*: Interdental regions where the imaginary lines passing through the cemento-enamel junctions of two adjacent teeth and the alveolar bone crest were parallel to each other and where the alveolar bone level was in a more apical position than it should be in the healthy periodontium were labeled with the horizontal bone loss label [12–14]. During the segmentation process, the cemento-enamel junctions of two adjacent teeth, the most distal border of the mesial tooth, the alveolar crest line in the relevant interdental region, and the most mesial border of the distal tooth were followed, and the specified reference areas were combined in the form of a closed curve. When the relevant labels were completed, all of the segmentation areas resembled geometric shapes such as squares, rectangles, and trapezoids (Fig. 1-b).

*For vertical bone losses*: Two imaginary lines were created from the cemento-enamel junctions of two adjacent teeth and the alveolar bone crest line. The interdental regions where these two lines are at an angle to each other were labeled with the vertical bone destruction label [12–14]. When completed, the labels showed a triangular geometry. Minimal ridge angles due to changes in the tooth axis are not included in this label. The criteria specified for horizontal bone loss were followed when creating labeling boundaries (Fig. 1-b).

*For furcation defects*: The unit of this parameter was the tooth. Furcation areas of multi-rooted teeth were examined and radiolucent images compatible with bone resorption were labeled with the furcation defect label. While labeling was carried out, the root boundaries of the relevant tooth and the boundaries of the radiolucent region formed by the lesion were followed and completed to form a closed curve (Fig. 1-b).

After labeling was completed, the data set was rechecked by the observers. At this stage, instead of reviewing the boundaries of segmentation, the bone resorption form and geometry of the labeled region were quickly reviewed to avoid standardization deficiencies that may arise in horizontal-vertical bone loss decisions. In addition, consensus was reached in cases where dentists were conflicted about labeling some initial furcation defects. Also, the final evaluation made by 4 dentists together allowed the forgotten and overlooked labels to be noticed and completed before AI training. Labels with no consensus on diagnosis were removed and not used in algorithm training and result metrics.

### Model training

**Fig. 2:**
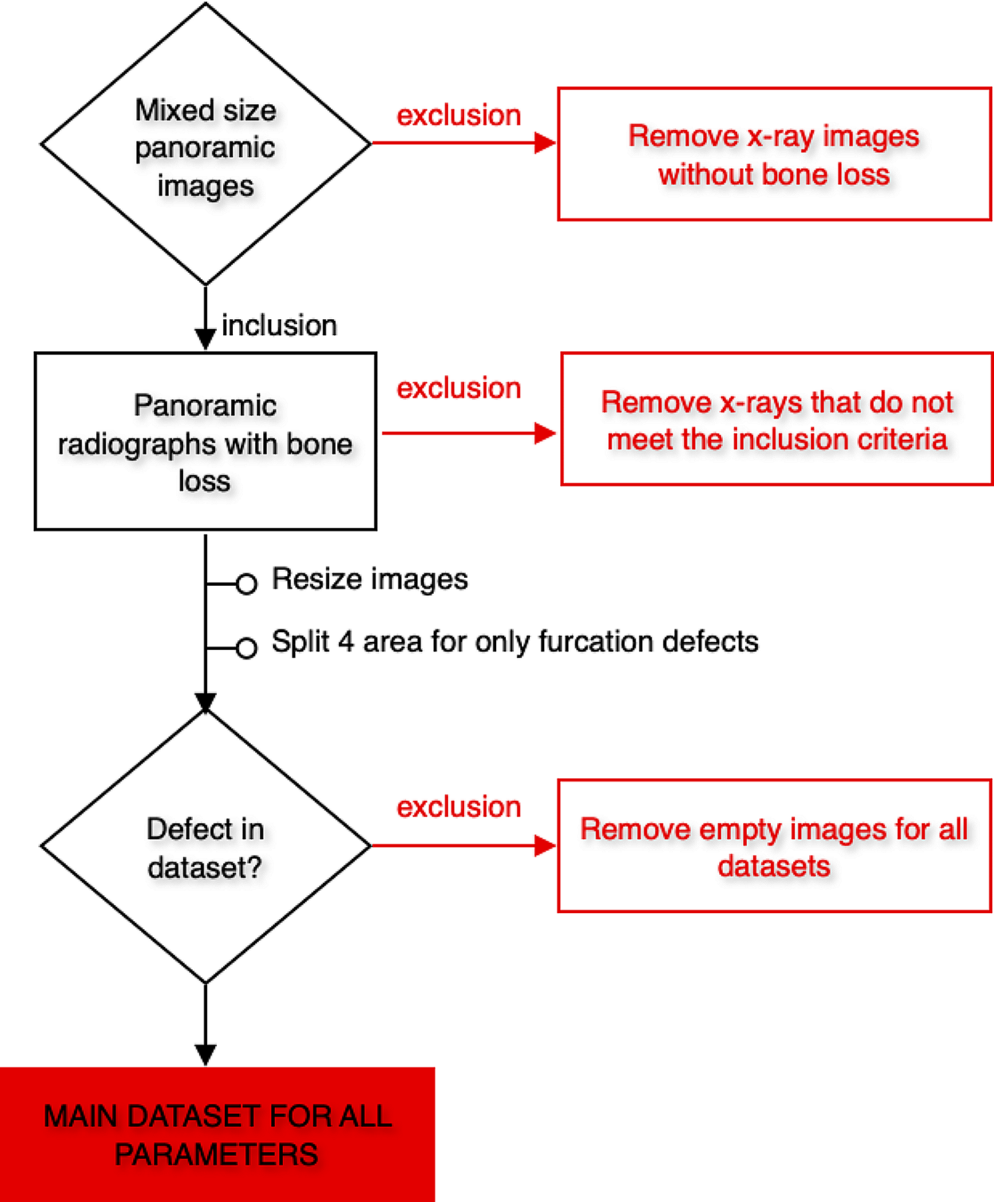
Workflow demonstrating the application of inclusion and exclusion criteria

**Fig. 3:**
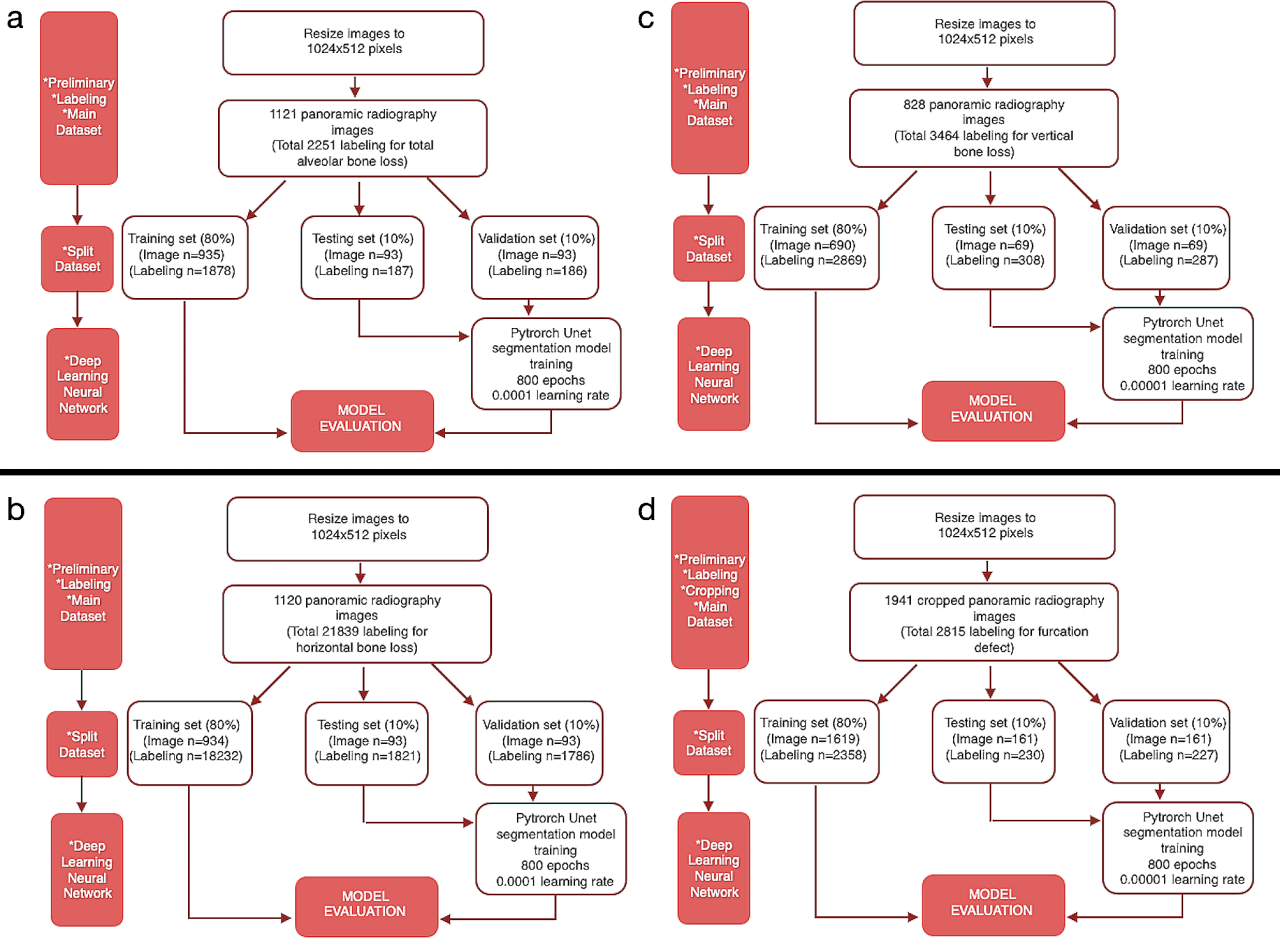
The stages of creating the datasets to be used in the development of AI models for all parameters. (**a**) Total alveolar bone loss; (**b**) Horizontal bone loss; (**c**) Vertical bone loss; (**d**) Furcation defect

*1. Pre-processing steps*: All images were resized as 1024 × 512 pixels. Panoramic radiographs to be used in the data sets created for developing the furcation defect model were cropped to 4 (left mandible, right mandible, left maxilla, and right maxilla). Since furcation defects were only seen in multi-rooted molars, it was aimed to focus more on the relevant areas in this way. The radiographs to be used in other parameter models were not cropped but used as a whole. Images that do not have the related parameter label were excluded from the main data sets of that parameter (Figs. 2 and 3). In line with these criteria, 1121 panoramic radiographs for total alveolar bone losses, 1120 panoramic radiographs for horizontal bone losses, 828 panoramic radiographs for vertical bone losses, and 1941 cropped panoramic radiographs (890 panoramic radiographs) for furcation defects were included in the main datasets (Table 1; Fig. 3).

**Table 1:** Numerical data on the development of deep learning algorithms

Model Name	Number of Training Images	Number of Training Labels	Number of Validation Images	Number of Validation Labels	Number of Test Images	Number of Test Labels	Epoch	Learning Rate	Model
**Total Alveolar Bone Loss**	935	1878	93	186	93	187	800	0.0001	U-Net
**Horizontal Bone Loss**	934	18,232	93	1786	93	1821	800	0.0001	U-Net
**Vertical Bone Loss**	690	2869	69	287	69	308	800	0.00001	U-Net
**Furcation Defect**	1619	2358	161	227	161	230	800	0.00001	U-Net

*2. Training, Validation, and Testing Data*: The main datasets were created by combining all panoramic radiographs containing the relevant parameter labels for each parameter. Then these main datasets were split as a training set (80%), validation set (10%), and testing set (10%) randomly for the development of AI systems (Table 1).

Training set: 935 images (1878 labels) for total alveolar bone loss, 934 images (18232 labels) for horizontal bone loss, 690 images (2869 labels) for vertical bone loss, and 1619 cropped images (2358 labels) for furcation defect were used as training data-set.

The validation set: 93 images (186 labels) for total alveolar bone loss, 93 images (1786 labels) for horizontal bone loss, 69 images (287 labels) for vertical bone loss, and 161 cropped images (227 labels) for furcation defect were used as validation data-set.

Test set: 93 images (187 labels) for total alveolar bone loss, 93 images (1821 labels) for horizontal bone loss, 69 images (308 labels) for vertical bone loss, and 161 cropped images (230 labels) for furcation defect were used as testing data-set.

The labels in the training set were used to create the algorithm of the AI model. This model was tested with the data in the validation set for adjusting the algorithm and questioning the need for further training. The final version of the algorithm, which was developed using the training set and successful trials were carried out with the labels in the validation set, was used in the detection of periodontal bone destruction and pattern on the radiographs in the testing set. The diagnostic results of the system in the testing set were compared with the observer labels in this dataset by a computer command, and the success metrics of the system were presented. In short, while determining the final success of the model, the observer diagnoses in the test dataset were accepted as the gold standard and these labels were used as the ground truth dataset. (Fig. 3). These steps were performed separately for each parameter.

**Fig. 4:**
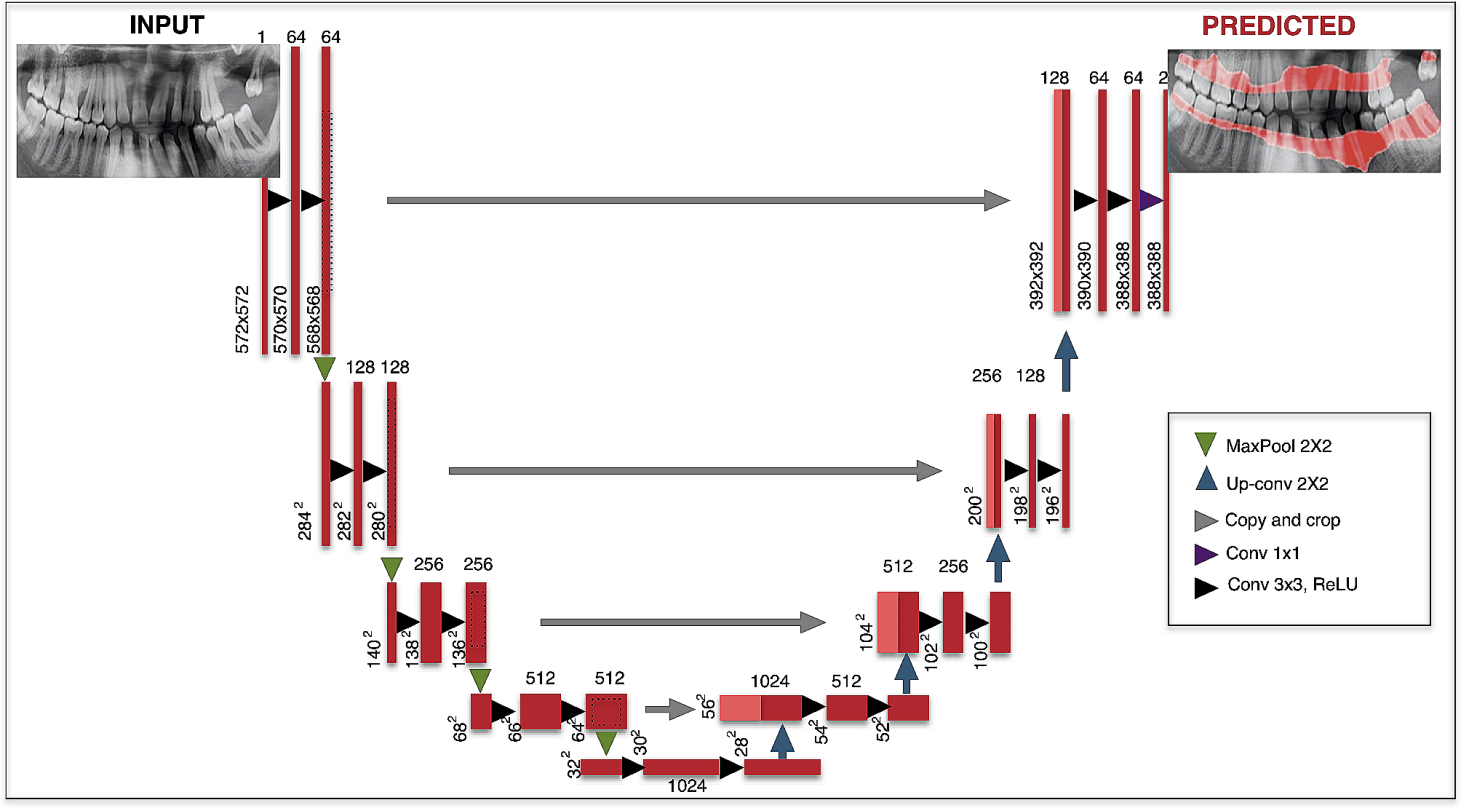
U-Net architecture and deep learning stages. Red tiles were used to show the multichannel feature map and light tiles were used for the copied feature map. Different colored arrows represented different processes in the architecture [34]

*3. Description of CNN architecture*: The U-Net architecture was used to perform deep learning (Table 1) [24, 34]. U-Net is a type of CNN that can perform semantic segmentation assignments on various images such as radiography images. U-Net contains four block levels with 32, 64, 128, and 256 convolution filters in each block. The working mechanism of this architecture is to convert images to vectors for pixel classifications and then convert these vectors back to images for segmentation. The encoder and decoder paths are used in order and perform model training. There is a maximum pool layer in the paths of encoders and up-convolution layers in the paths of decoders. For the current study, the U-Net architecture steps were followed and the working mechanism was presented in Fig. 4 in detail [24, 34].

*4. Method for training*: PyTorch library (v. 3.6.1; Python Software Foundation, Wilmington, DE, USA) and Adaptive Moment Estimation (ADAM) optimizer (for updating the learning rate for each parameter) were used in the model development stages. All training was made with a computer equipped with 16 GB RAM and NVIDIA GeForce GTX 1660 TI graphics card. U-Net architecture was used in the training of all parameters and all of them were trained with 800 epochs. Training of total alveolar bone loss and horizontal bone loss parameters was carried out with a learning rate of 0.0001, and training of vertical bone loss and furcation defect parameters was carried out with a learning rate of 0.00001.

### Assessment metrics

The statistical phase of the study was carried out with the confusion matrix method and ROC analysis. A script was created to perform these evaluations automatically with the help of a computer. A computer program (Python version 3.6.1, Python Software Foundation) and OpenCV and NumPy libraries were used to develop this script. The script used at this stage accepted the labels made by experienced dentists in the testing set as the gold standard. It automatically presented a success metric by comparing the diagnostics of the algorithm in the relevant dataset with the observers’ diagnoses. While the script was running, it presented results using some calculations below.

Sensitivity, precision, and F1 scores were calculated using the confusion matrix method [24]. Initially, three different calculations were for the confusion matrix method: numbers of true positives (TP, there was a periodontal problem and it was segmented correctly), numbers of false positives (FP, there wasn’t a periodontal problem but it was detected wrongly), and numbers of false negatives (FN, there was a periodontal problem but it wasn’t detected). Then, sensitivity, precision, and F1 scores were determined with the following calculations:

Sensitivity (recall): TP/ (TP + FN).

Precision: TP/ (TP + FP).

F1 score: 2TP/ (2TP + FP + FN).

In addition to the confusion matrix method, receiver operating characteristic (ROC) curve analysis and precision-recall evaluation were also performed to provide more detailed data. The area under the ROC curve (AUC) values are calculated by this analysis. ROC is a probability curve and the area under it, AUC, represents the degree or measure of separability. As the area under the curve increases, the discrimination performance between classes increases. It is known that as the success of the system increases, the AUC grows and approaches the value of 1. In line with this information, interpretations were made and the results were presented. In addition, the labels and system results of the radiographs in the test data set in which the evaluations were carried out were reviewed by experienced dentists for each parameter.

## Results

### Study data

**Fig. 5:**
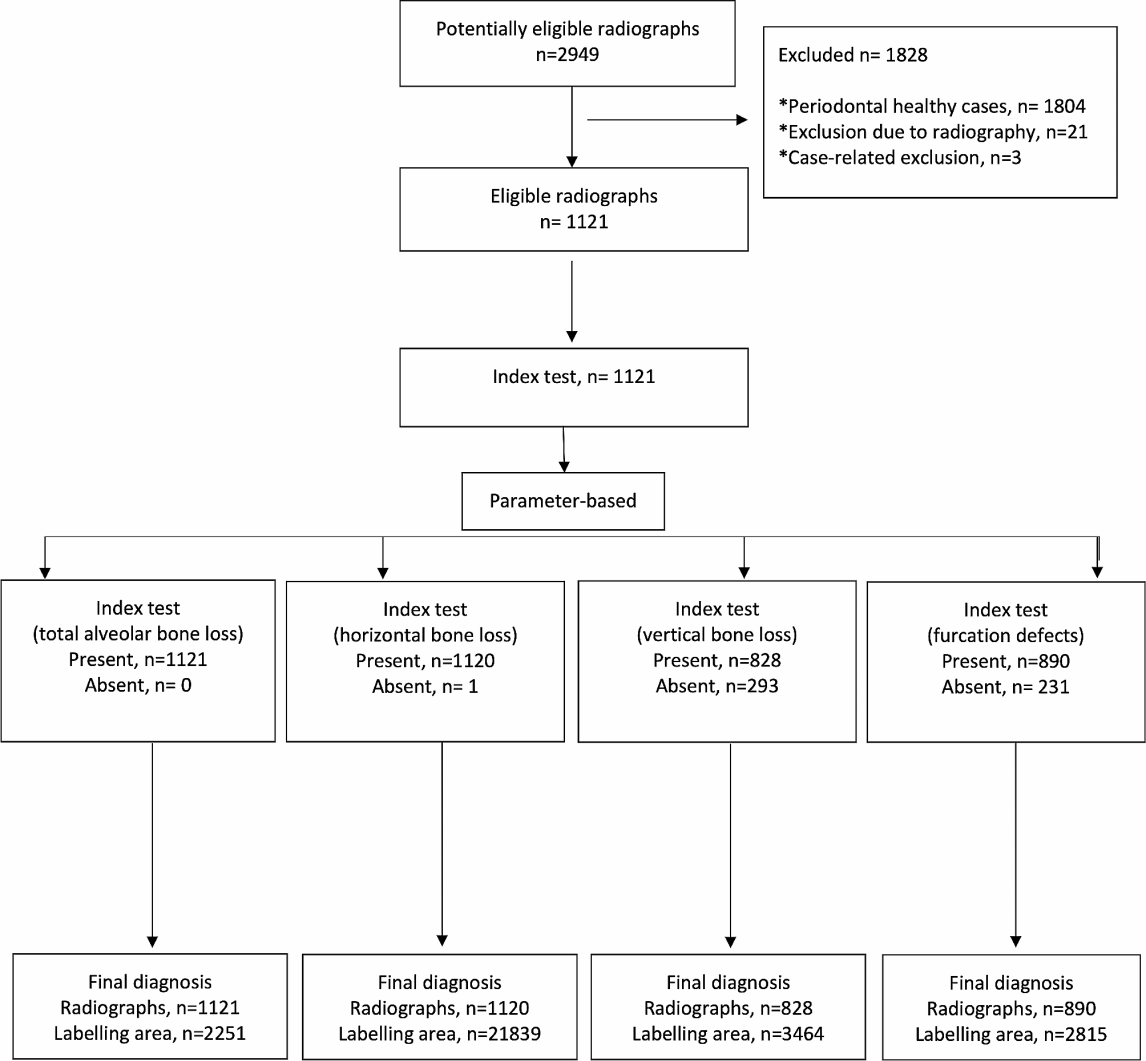
Standards for Reporting Diagnostic Accuracy (STARD) flow diagram

A total of 2949 radiographs were examined in the study. 1804 of them were excluded from the study because they were not periodontitis cases. In addition, 24 of the remaining images were excluded from the study due to radiography (*n* = 21) and case-based reasons (*n* = 3). Observers agreed on the periodontitis diagnosis of 1121 panoramic radiographs. These radiographs contained a total of 30369 labels as 2251 total alveolar bone loss, 25303 interdental bone losses (21839 horizontal bone losses, 3464 vertical bone losses), and 2815 furcation defects in total (Fig. 5). The total number of labels presented for each parameter indicated the presence of pathology related to the relevant parameter (bone losses or furcation defects). If the relevant pathology was not present, a label was not available in the relevant area.

### Intra-observer and inter-observer agreements

It was found that each investigator showed high consistency in the diagnosis and segmentation of these parameters at different times (IoU > 0.91). In addition, kappa values were calculated to evaluate the consistency of the observers’ decision for horizontal and vertical bone losses in the interdental regions. Kappa values showed excellent agreement when the two times were compared in the decision of bone loss characteristics in each investigator (K = 0.81–0.99). Also, the labeling areas of different observers were examined for evaluation of inter-observer agreement using this data set. The IoU values obtained were found to be between 0.8126 and 0.8737. While the results were 0.85 and above for horizontal bone loss, total alveolar bone loss, and furcation defect, they were lower for vertical bone loss (> 0.81).

### Outcomes

While the confusion matrix evaluates a given classifier with a fixed threshold, the AUC evaluates that classifier over all possible thresholds. Therefore, AUC was considered as the primary outcome. AUC values were 0.951 for total alveolar bone loss, 0.910 for horizontal bone losses, 0.733 for vertical bone losses, and 0.868 for furcation defects (Table 2). The developed model showed the most successful results when determining total alveolar bone loss (AUC = 0.951). The model’s lowest success rate was seen when detecting vertical bone loss (AUC = 0.733).

**Table 2:** The results obtained with the confusion matrix method and ROC analysis

Model Name	Found Correct for IoU Threshold: 50%	Found Wrong for IoU Threshold: 50%	Not Found for IoU Threshold: 50%	Sensitivity	Precision	F1 Score	Accuracy	AUC
**Total Alveolar Bone Loss**	185	1	0	1	0.995	0.997	0.994	0.951
**Horizontal Bone Loss**	108	7	6	0.947	0.939	0.943	0.892	0.910
**Vertical Bone Loss**	115	21	91	0.558	0.846	0.673	0.506	0.733
**Furcation Defect**	181	13	22	0.892	0.933	0.912	0.837	0.868

**Fig. 6:**
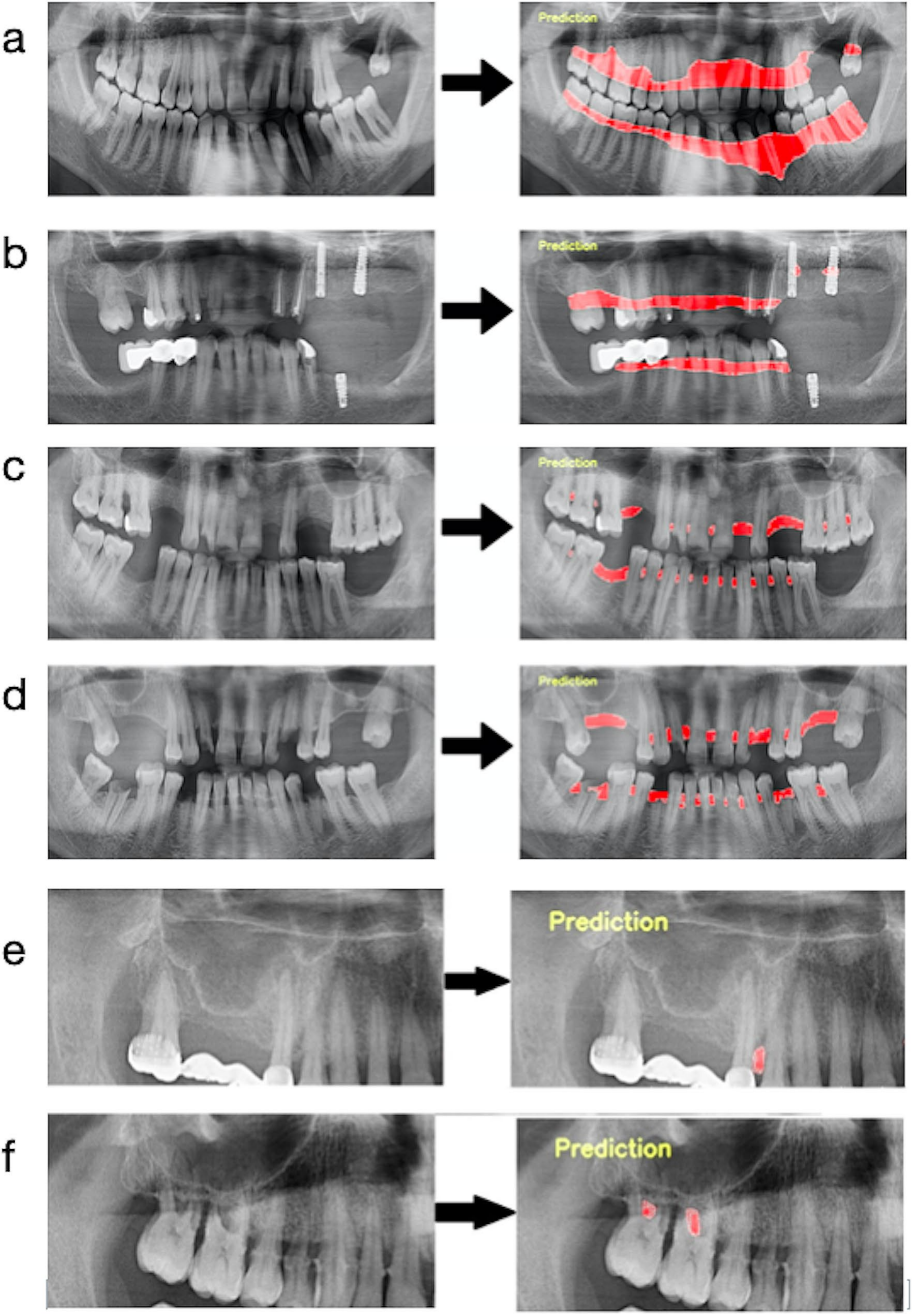
Input and output data of some radiographs of patients. **a-b**. Total alveolar bone loss; **c-d**. Horizontal bone loss; **e**. Vertical bone loss, **f**: Furcation defect

The results were calculated with the confusion matrix method, which demonstrates the success of the developed AI model, were presented in Table 2. When the diagnostic performance metrics were evaluated, sensitivity, precision, F1 score, and accuracy values, respectively, were 1, 0.995, 0.997, 0. 994 for total alveolar bone loss; 0.947, 0.939, 0.943, 0.892 for horizontal bone losses; 0.558, 0.846, 0.673, 0.506 for vertical bone losses and 0.892, 0.933, 0.912, 0.837 for furcation defects at 50% IoU. In Fig. 6, The input and output images of the radiographs of some patients in the AI system were shown as an example.

**Fig. 7:**
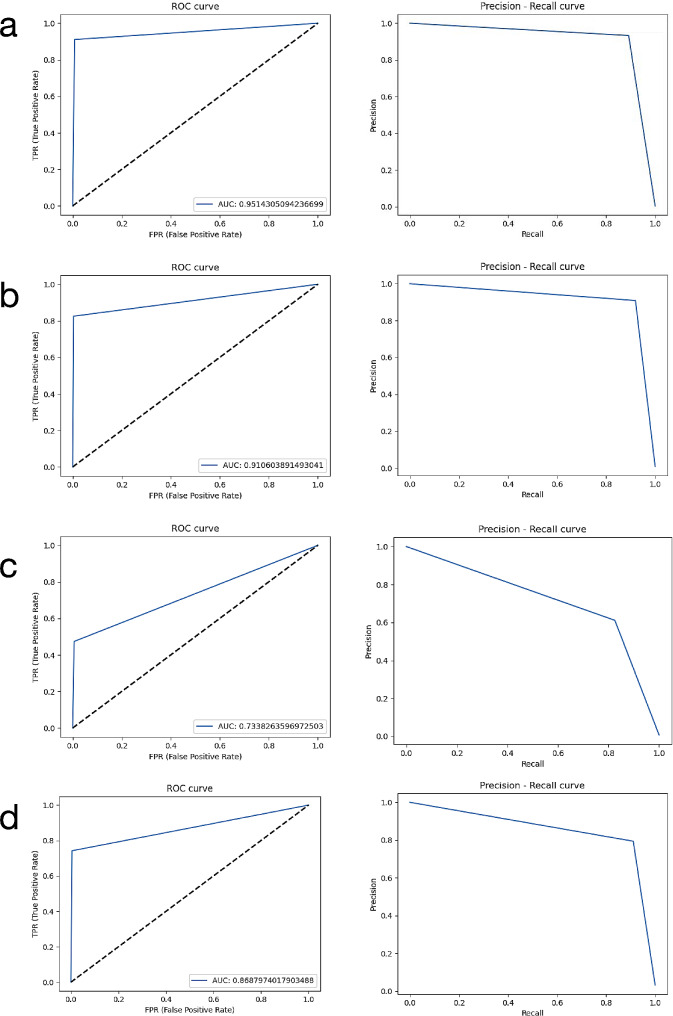
ROC curve analysis and precision-recall results of all parameters. (**a**) Total alveolar bone loss; (**b**) Horizontal bone loss; (**c**) Vertical bone loss; (**d**) Furcation defect

Also, ROC curve analysis and precision-recall results for all periodontal parameters were presented in Fig. 7.

### Observations

In addition, the input and output images of each case in the testing set were reviewed by the observers who labeled them, and it was analyzed in which situations the system made more mistakes. When the output images of total alveolar bone loss were examined, it was observed that the success of the system was quite good, and there were few errors in the detection of only some deep angular bone loss areas, and furcation areas. When the output images of horizontal bone loss were examined, it was analyzed that the system could not diagnose in some radiographs in large interdental regions caused by tooth deficiency. It was observed that some vertical bone losses were determined as horizontal bone loss in regions with partial superimposition of anatomical structures. The most common error made by the system in detecting vertical bone losses was to perceive very minimal bone angulations as vertical bone losses. The system identified some of the bone losses in the interdental regions of roots close to each other as furcation defects. Another mistake was that the AI model overlooked and failed to detect some of the initial level furcation defects.

## Discussion

In recent years, the use of AI systems in medicine and dentistry in image interpretation has attracted great interest and CNN systems change these areas rapidly [24]. However, the number of studies conducted in the field of periodontology is still limited. The current study aims to automatically evaluate panoramic radiographs by an AI system and to determine the success of the related system in detecting periodontal disease findings on radiographs. The results of this study showed that AI systems can be a decision-support mechanism for dentists in the diagnosis of periodontal disease, one of the most common diseases in the world.

Different architectures can be used in AI-based studies. U-Net architecture, a CNN-based AI architecture, is one of the image segmentation techniques that enable the evaluation of images in the medical field with AI systems and provides a more precise and successful evaluation with fewer training sets [35, 36]. Therefore, in the current study, image processing was performed using the U-Net architecture.

In the literature, there were many studies in which AI algorithms were used in the processing and interpretation of patient data, which played a role in diagnosis and treatment planning, such as dental radiographs [20, 37–39], intraoral photographs [40–43] and pathology images [44]. In addition, these systems were also used in situations such as pre-examination evaluation and risk estimation [45]. Also, it was seen that some of the studies aiming to determine periodontal disease from 2D dental radiographs using AI systems were performed on periapical radiographs [21, 28, 46] and some on panoramic radiographs [3, 6, 25, 29, 30].

In one of the studies in the literature, Lee et al. (2018) used 1740 periapical radiographs and evaluated the prediction success of the CNN algorithm they developed. In the results of this study, they reported that the accuracy of determining premolar teeth with periodontal damage was 81% and 76.7% for molars [27]. Similar to this study, Lee et al. (2021) performed another study on 693 periapical radiographs [18]. In this study, it was aimed to find the radiographic bone loss by grouping it according to the severity of destruction (stages I-II-III) [18] High success rates were found in the detection of periodontal disease in the results of these two studies.

On the other hand, Khan et al. (2021) tried to find dental problems such as caries, bone losses, and furcation defects on 206 periapical images using different AI architectures (U-Net, X Net, and Seg Net) in their study [28]. Although this study was similar to our study in terms of using the segmentation method in the detection of periodontal bone loss, no evaluation was made in terms of bone destruction characteristics (horizontal, vertical) [28]. Another difference was Khan et al. (2021) performed their studies on periapical radiographs and with fewer data [28]. Although the evaluations were made on different types of radiographs, it could be said that the success rates of our study were higher. We think that the reason for the higher success rates of the AI system developed in our study may be due to the use of a larger data set. Because as the number of data used for training in AI studies increases, the success rates of the model also increase [47].

In another study, Kurt Bayrakdar et al. (2021) used Google Net Inception v3 architecture on a large data set (2276 panoramic radiographs). They evaluated the success of the AI systems they developed in the determination of radiographs of periodontitis cases [6]. In this study, 1137 radiographs of patients with bone loss and panoramic radiographs of 1139 periodontal healthy individuals were used for the development of the AI model. F1 score and accuracy were found as high as 91% in this study [6]. However, no labeling was made, only the classification method was used to train the radiographs in the form of patient/healthy and to find them by the system. In this respect, they are quite different from our study in terms of this study planning. Based on this information, it can be said that the segmentation technique we used in our study is the most advantageous method and provides the physician with more detailed information for diagnosis and treatment planning.

There were also studies in the literature comparing the diagnosis of dentists with different experiences and AI model predictions. The results obtained in this way are undoubtedly more interpretable and reveal the success of the system more clearly. For example, Krois et al. (2019) compared the evaluation of 6 dentists and the results of AI in a panoramic radiography study with the CNN technique. They reported the accuracy, specificity, and sensitivity rates for the system as 81% in the results of their study [25]. Since this study deals with the evaluation of many dentists, it was a more comprehensive and superior study in terms of planning. However, in the study of Krois et al. (2019), periodontal bone loss patterns were not classified as horizontal or vertical.

In another similarly planned study, Kim et al. (2020) compared the evaluations of 5 different clinicians with the performance of AI in their study for the determination of bone resorption sites using 12179 panoramic radiography [29]. They reported that while the average F1 scores of the clinicians’ results in determining bone loss were 69%, AI showed higher success and the F1 score was 75%. This study reveals the success of AI systems in radiography interpretation and shows promise for the future use of these systems [29]. Also, Chang et al. (2020) tried to perform the determination of bone loss from panoramic radiographs without any evaluation of bone destruction angulation and defect type [3]. In this study, the staging was tried to be made according to the 2017 periodontitis classification by calculating the amount of bone destruction and destruction [3].

Finally, Jiang et al. (2022) tried to detect periodontal bone destruction in their study using the CNN model using 640 panoramic radiographs. This study is the most similar to our study in the literature [30] because Jiang et al. (2022) also determined the bone loss patterns of periodontal disease in the form of vertical/horizontal/furcation defects in their study [30]. Although it was similar to our study in this respect, the object detection method was used in this study for labeling. The segmentation method we use is a much more advantageous method since it determines the defective area with its borders. Because it provides more detailed information to the dentist to determine the severity of the disease and to plan the treatment in the next process. In addition, in this method, the defect area is processed like a detailed map and provides more advanced diagnostic support visually. On the other hand, when two studies were compared in detail, it was seen that they used multiple observers, and two different AI architectures (U-Net and Yolo-v4), and included the severity of the disease in their assessment.

One of the limitations of the study was that the decision of multiple observers was not separately compared with the predictions of AI. Another limitation was that no measurements were made to determine disease severity in the study and it was aimed to detect only bone losses. More extensive research could have been done using calibrated panoramic radiographs, based on direct measurement, or to determine the percentage of root affected. However, It should be noted that in all 2D radiographic imaging techniques, the evaluation of bone craters, lamina dura, and periodontal bone level is limited to the projection geometry and superposition of adjacent anatomical structures [48]. In other words, these radiographs do not provide clear and reliable information for measurement and treatment planning. For this reason, performing AI-based studies with three-dimensional (3D) radiographic imaging techniques such as cone-beam computed tomography systems (CBCT) will prevent this limitation. Undoubtedly, as the number of studies in this field increases, AI algorithms with stronger decision-support capability and providing much more detailed information will be developed. Despite the limitations, our study is promising for such studies to be carried out in the future. In addition, the performance of the AI model in determining vertical bone losses in our study was lower than other periodontal parameters. It is a known fact that vertical bone losses are less common than horizontal bone loss, and therefore, the number of labels for vertical bone losses was more limited in our study [14]. We think that the number of labels used in the detection of vertical bone losses with AI in our study was therefore less, and this situation caused the performance of the model to be found to be lower. Because the most important thing in the success of AI studies is to work with large data sets. This limitation could be overcome, albeit to a limited extent, by using different AI architectures and making different technical plans. For example, in our study, cross-validation techniques could be used during model development and multi-class training could be done. On the other hand, the more important reason for the low success rates for the vertical bone loss parameter also may be the incomplete understanding of the outline of the alveolar bone crest in 1, 2, and 3-walled defects. We think that interobserver agreements were also lower for this parameter due to this limitation, which may be due to the limited view of panoramic radiography. This diagnostic difficulty can be eliminated by using some radiography techniques that provide more detailed images in future studies.

## Conclusions

When the literature was examined, it was seen that the use of AI systems in dental radiographs for periodontal status determination has very successful and promising results. To the best of our knowledge, the present study is the first study aimed at detecting bone resorption and its patterns using deep learning algorithms and segmentation methods. These bone loss patterns play an important role in periodontal treatment planning and the prognosis of teeth. With this aspect, these systems are candidates to be a decision-support mechanism for dentists in radiographic interpretation. The development of these systems with more data sets will increase success rates. There is a need for more comprehensive studies on 2D and 3D radiographs in this regard.

## Data Availability

The datasets generated and/or analyzed during the current study are not publicly available due to the conditions specified when receiving approval from the ethics committee but are available from the corresponding author upon reasonable request.

## Abbreviations

CNN: Convolutional Neural Network
AI: Artificial intelligence
2D: Two-dimensional
IoU: Intersection over Union
ADAM: Adaptive Moment Estimation
TP: Numbers of true positives
FP: Numbers of false positives
FN: Numbers of false negatives
ROC: Receiver operating characteristic
AUC: Area under the ROC curve
3D: Three-dimensional
CBCT: Cone-beam computed tomography systems
STARD: Standards for Reporting Diagnostic
